# The emergence of social soft skill needs in the post COVID-19 era

**DOI:** 10.1007/s11135-023-01659-y

**Published:** 2023-04-17

**Authors:** Giorgio Gnecco, Sara Landi, Massimo Riccaboni

**Affiliations:** 1 Scuola IMT Alti Studi Lucca, Piazza S. Francesco, 19, Lucca, Italy; 2 LUISS University, Viale Romania, 32, Rome, Italy

**Keywords:** Counterfactual analysis, COVID-19, Matrix completion, Work from home, Soft skills, C44, C53, C63, J24, J44

## Abstract

Social soft skills are crucial for workers to perform their tasks, yet it is hard to train people on them and to readapt their skill set when needed. In the present work, we analyze the possible effects of the COVID-19 pandemic on social soft skills in the context of Italian occupations related to 88 economic sectors and 14 age groups. We leverage detailed information coming from ICP (i.e. the Italian equivalent of O*Net), provided by the Italian National Institute for the Analysis of Public Policy, from the microdata for research on the continuous detection of labor force, provided by the Italian National Institute of Statistics (ISTAT), and from ISTAT data on the Italian population. Based on these data, we simulate the impact of COVID-19 on workplace characteristics and working styles that were more severely affected by the lockdown measures and the sanitary dispositions during the pandemic (e.g. physical proximity, face-to-face discussions, working remotely). We then apply matrix completion—a machine-learning technique often used in the context of recommender systems—to predict the average variation in the social soft skills importance levels required for each occupation when working conditions change, as some changes might be persistent in the near future. Professions, sectors, and age groups showing negative average variations are exposed to a deficit in their social soft-skills endowment, which might ultimately lead to lower productivity.

## Introduction

Soft skills are often referred to in the literature as interpersonal, human, people, or behavioral skills and rely on personal behavior (Lee 2019). They are typically measured based on surveys (Deming 2017). The value of soft skills in the workplace has been documented for decades, and the current literature emphasizes the importance of soft skills as complementary to hard skills, i.e., those related to scientific and technical knowledge (Hendarman and Cantner 2018). Examples of social soft skills include: cooperating; listening actively; monitoring; taking care of others. According to the results of a recent survey (Lamberti et al. 2021), the development of soft skills during higher education has been perceived as highly relevant by students who were employed later in high-salary jobs. Among soft skills, soft skills that involve interaction with other people (i.e. social soft skills) are expected to have been significantly affected by the COVID-19 pandemic, due to its induced changes in working conditions. Social soft skills are very important when working in teams because they can have a significant effect on team performance and on how positively a worker is received by the other components of a team.

The literature on the effects of COVID-19 on social soft skills is still quite limited. According to Brucks and Levav (2022), the increase of virtual interaction and work from home induced by COVID-19 may have inhibited social soft skills because in-person teams have the possibility to discuss their ideas in the same fully shared physical place. In contrast, virtual teams have a more constrained interaction, bounded by the presence of a screen in front of each team component. Moreover, Melin and Correll (2022) find positive effects (according to participants’ self-assessment of their social soft skills) of an online intervention program (consisting of virtual peer groups and online career coaching) aimed at developing social soft skills among early-career women in a North-American firm during the pandemic.

This work aims to fill the research gap on the relationship between COVID-19, remote work and social soft skills, by investigating the possible effects of the COVID-19 pandemic on social soft skills, focusing on Italy as a case study. This goal is achieved by exploiting a variety of data sources—some statistics about the Italian working population provided by the Italian National Institute of Statistics (ISTAT) and the results of the Italian Survey on Occupations (ICP, Indagine Campionaria sulle Professioni), which provide, among others, measures of average importance levels of social soft skills across different professions—and applying a supervised machine-learning technique (matrix completion) to see how its predictions of average importance levels of social soft skills change by considering different simulated post-COVID-19 scenarios. The relevance of this analysis stems from the fact that some changes might be persistent in the near future due to hybrid and remote work. Hence, professions, sectors, and age groups for which negative (average) variations are predicted for specific social soft skills are exposed to a deficit in their endowment of social soft skills, which might ultimately lead to undesirable effects such as lower productivity.

The choice of Italy as a case study derives both from the availability of data for our analysis and from the fact that the COVID-19 emergency expanded extremely rapidly in Italy, inducing the Italian government to adopt serious economic and social countermeasures to preserve public health, such as locking down several industrial sectors (Baldwin and Di Mauro 2020). In this severe situation, workers employed in sectors that require physical proximity to customers or colleagues and those exposed to diseases and infections were the most at risk. For most other categories, it was still possible to keep performing their daily job working from home. The Italian legislative setting was modified in 2017 with Law 81 to foster remote working. In this framework, Italy is an interesting environment to carry out our study: the country’s labor market is characterized by high rigidity in work organization, but recently firms began to express interest in remote working, even if before the pandemic, this approach remained confined to a small number of working categories.

Social distancing was essential for addressing the COVID-19 crisis, as it reshaped the landscape of economic activities, with a heterogeneous impact across occupations. Specifically, nonessential jobs characterized by a high degree of physical interaction suffered the most because consumers reduced their demand due to social distancing. Similarly, essential workers were compelled to remain in their workplaces, increasing the risk of contagion among them. At the same time, the possibility of carrying out some of their work from home allowed them to absorb the negative effects of the lockdown partially. The difficulties faced during the COVID-19 emergency, particularly during the lockdown, have been a stress test for social soft skills. Among these skills, adaptability, communication skills, empathy and relationship building suffered the most and needed much attention from the employers’ and employees’ perspectives. Focus on social soft skills is motivated by the fact that such skills already played an increasingly important role in the job market before the pandemic. Still, after the COVID-19 crisis, their demand is expected to increase even further.

In our analysis of the possible effects of the COVID-19 pandemic on social soft skills in Italy, we make use of the data coming from the results of the ICP survey, which represent, for every profession, the importance level (averaged over the respondents) of each skill, competence, working attitude, working style, generalized working activity, and working condition in Italy. These data are collected in a matrix, denoted in the article as ICP matrix, in which the rows refer to the professions. In contrast, the columns refer to the answers to questions in the survey related to specific skills, competencies, working attitudes, working styles, generalized working activities, and working conditions.^1^ The ICP survey contains variables that are extremely useful to illustrate the potential risks workers faced during the COVID-19 emergency, as well as to formulate hypotheses and make predictions on how the labor market will move on in the near future. Following Barbieri et al. (2022), by examining the columns of the ICP matrix, we identify five among the working conditions considered in the ICP survey that were mostly affected by the spread of the pandemic and by the related consequent countermeasures. We then create three possible post-COVID-19 scenarios based on how strongly the pandemic affected the above conditions: 25% (low), 50% (medium), and 75% (high). In each of such scenarios, we reduce or increase the values of the elements of the corresponding five columns associated with the selected working conditions in the original ICP matrix, thus obtaining a modified (or perturbed) ICP matrix, whose relevance derives from the fact that it represents the direct effect of the associated simulated post-COVID-19 scenario on those columns. Then, for the analysis of such matrices, we apply to each of them a supervised machine-learning technique: namely, Matrix Completion or MC (Mazumder et al. 2010).^2^ Such a technique allows one to predict (or reconstruct) a subset of elements of a matrix based on the observation of another subset of its elements. It is commonly applied as a state-of-the-art technique, e.g. in the context of recommender systems, to predict user’s preferences, as in the case of item ratings (a famous example in the related literature being the case of movie ratings, see Hastie et al. (2015)). In the present study, the aim of the MC application—which justifies its choice for the analysis—is to predict average importance levels of social soft skills for each profession based on a subset of other elements of each modified ICP matrix (i.e. by considering different simulated post-COVID-19 scenarios). The same MC approach was applied with success (i.e. showing excellent prediction accuracy) in Gnecco, Landi, and Riccaboni (2022) to analyze the average importance levels of soft skills for creativity. In that work, however, no matrix perturbation induced by a simulated post-COVID-19 scenario was considered (i.e. MC was applied therein not to modified ICP matrices but only to the original ICP matrix). Another difference is that the present work is focused on the analysis of a different set of soft skills (namely, on the analysis of quite a large set of social soft skills). Additionally, in the present study, we compare the MC predictions of average importance levels of social soft skills in each simulated post-COVID-19 scenario with the corresponding MC predictions in the baseline scenario to assess the impact of each simulated scenario on the social soft-skills endowment of each profession. Finally, to derive the implications of our analysis of social soft skills endowments across sectors and workers’ age groups, we combine the results obtained by MC with the Microdata for Research (MFR) on the Continuous Detection of Labor Force (RCFL)^3^ provided by the Italian National Institute of Statistics (ISTAT)—which gives us the economic sector and activity workers are associated with, and their age group—and with ISTAT data on the Italian working population. It is worth noticing that no combination of the ICP dataset with the MFR RCFL dataset and with ISTAT data on the Italian population was performed in the previous work Gnecco, Landi, and Riccaboni (2022). We show that among selected social soft skills, cooperating, managing working groups, coordination with others, teamworking, and teaching are among the most negatively impacted in the simulated post-COVID-19 scenarios (i.e. the ones experiencing the most negative decreases of MC predictions of average importance levels of social soft skills), whereas a positive impact is obtained only for consultancy. Moreover, macro-sectors (ATECO sections) related to commercial activities, tourism, and education are among the most negatively impacted ones in the simulated post-COVID-19 scenarios, whereas the most negatively impacted age groups refer to workers under 35 years old. These results and other findings obtained by MC at a more disaggregate level, are reported in Sects. 4.1 and 5.

The article is structured as follows. Section 2 reports related literature. Section 3 describes the datasets available for the analysis, whereas Sect. 4 illustrates the methodology adopted for that analysis. Section 5 summarizes our main results, whereas Sect. 6 provides some robustness checks. Finally, Sect. 7 concludes with a discussion.

## Related literature

This work builds on the existing literature on three main topics: soft skills, working from home, and matrix completion. In our work, these research topics are investigated by applying matrix completion to perturbed occupation matrices whose entries represent the average importance levels of soft skills for different jobs (a more detailed description is provided in Sect. 3). These perturbed occupation matrices are obtained by modifying some working conditions, such as working from home, according to various scenarios, which simulate various possible impacts of COVID-19.

First, our work contributes to the literature on soft skills. A soft skill can be broadly defined as knowledge in the human mind that is extremely personal, hard to formalize, and quite difficult to acquire naturally, as its development is based on personal experience (Lee 2019). One example of a soft skill (or, more precisely, a set of soft skills) is creativity, which is reviewed in Gnecco, Landi, and Riccaboni (2022). Indeed, soft skills are grounded in specific actions and experiences, which include emotionality, idealism, and values (Cirillo et al. 2021). Based on this premise, soft skills can be categorized as (inter)personal knowledge, i.e. knowledge obtained either by personal experience or from other individuals. For instance, the experience acquired by a teacher is surely rooted in conditions and situations that cannot be easily forecast, as teachers have their personalized experience (Amabile 1983; Mohajan 2016).

The term “soft skill” is used in the literature to highlight the contrast with “hard skill” (which refers to every skill related to scientific and technical knowledge). Comparing hard and soft skills, Laker and Powell (2011) observe that: (1) most people are able to distinguish between hard skills and soft skills; (2) training methods in hard and soft skills are typically different; (3) apart from entry-level positions, the majority of positions inside an organization require both hard and soft skills. Therefore, in the following, we focus on the important topic of measurement of soft skills. Currently, the measurement of soft skills is much less developed than the measurement of hard skills, for which specific tests were developed, e.g. the Intelligent Quotient (IQ) test. Indeed, hard skills are much better measured in terms of both reliability and validity. Nevertheless, it is still possible to measure soft skills based on specific survey questions (Deming 2017). According to Balcar (2014), two different approaches (direct and indirect) are typically used to measure soft skills. The direct approach is based on questioning people about their behavior or about their attitudes and preferences (i.e. respondents are asked to provide a self-assessment of their personality characteristics). Instead, the indirect approach takes the job tasks performed by an individual as proxies of soft skills. These are identified by experts or self-assessed by the worker. Typically, job tasks identified by the two categories of people (workers and experts) do not show significant differences. Recently, the Reading the Mind in the Eyes Test (RMET) was proposed as a novel way to measure soft skills (Deming 2017). Originally, it was developed with the aim of diagnosing “theory of mind” deficits such as autism (i.e. related to the capacity/incapacity to understand other people’s mental state). However, in a similar way as in the case of the IQ test for the measurement of hard skills, psychologists later discovered that the RMET has a significant predictive capability for a large variety of outcomes. In the present work, the measurement of soft skills is based on the results of a survey on a representative sample of Italian workers, as detailed in Sect. 3. A large number of questions (255) in that survey makes it possible for us to identify, among them, the ones that are specifically related to social soft skills.

Many researchers studied the occurrence and consequences of working remotely. The seminal article Oettinger (2011) analyzed how work from home grew in the period 1980–2000, as documented in the US census of population, and how this was related to changes in the frequency of face-to-face interactions, as addressed by the O*NET survey. Bloom et al. (2015) used a randomized controlled trial in the context of a Chinese travel agency for the estimation of the effects on productivity of home-based work. Mas and Pallais (2020) provided a review of the features and occurrence of alternative working arrangements (such as working from home) and their related demand. In that study, the authors reported data from the Quality of Worklife Survey and from the Understanding America Study and showed that a percentage smaller than about 13% of full- and part-time jobs had formal arrangements for smart working, even if more than 25% of workers often worked from home. According to the two scholars, the median worker claimed that only 6% of jobs could be feasibly performed from home, although several occupations (such as those related to mathematics, business and financial operations, and those involving the use of computers) could be carried out from home. Conversely, making use of the Skills Toward Employability and Productivity (STEP) survey on workers’ tasks, Saltiel (2020) measured the share of jobs that could be performed remotely and found that only a few jobs could be done from home, i.e. from 5 to 23% across the ten developing countries considered. The author’s analysis also demonstrated the presence of a positive correlation between the smart-working share and GDP per capita. In a deeper analysis of the characteristics of jobs that could be performed at home, Mongey et al. (2020) used O*NET data to build a measure of physical proximity within the workplace, for each occupation. Baker et al. (2020) and Koren and Pető (2020) used the same data to discover which occupations could not be done at home or would be negatively affected by social distancing. More recent research exploits surveys to measure smart-working in real-time (Brynjolfsson et al. 2020; McLaren and Wang 2020).

**Fig. 1:**
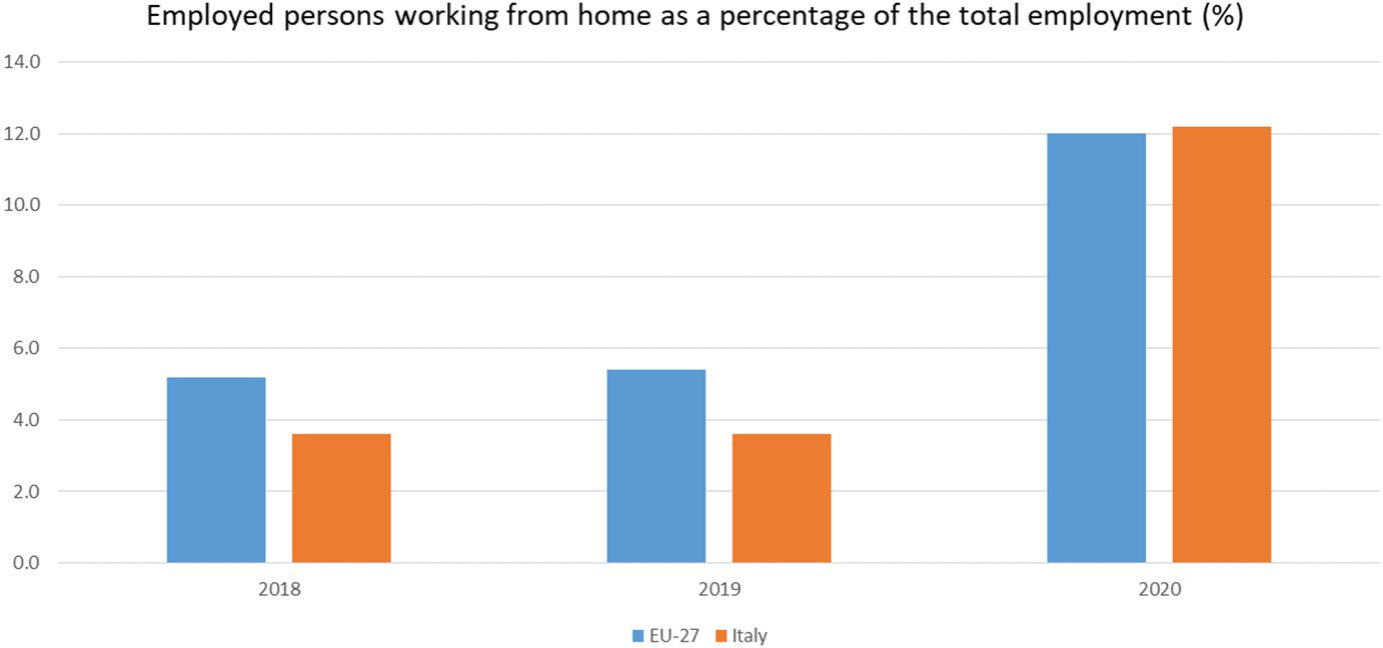
Telework in Italy vs. EU countries (% of total employment) *Source*: Labor Force Survey, 2021. (Color figure online)

Particular attention has been devoted to the concept of smart-working during the recent COVID-19 pandemic,^4^ a period that has demonstrated how more flexible working conditions are possible without necessarily affecting workers’ productivity negatively, and how much these flexible working conditions are often actually desired by workers, insofar as embracing them does not put remote workers at a disadvantage or negatively affects their well-being. In other words, the forced lock-down “experiment” that pushed masses of workers to work remotely at the same time has shown that more coordination and improved working relationships and thus efficiency gains are actually possible.^5^ In the US context, Brynjolfsson et al. (2020) documented that almost half of the people involved in their interview answered that they worked remotely in the first week of April 2020, whereas McLaren and Wang (2020) reported that about 35% of their US respondents worked entirely remotely in May 2020. The Decision Maker Panel, an entity set up by the Bank of England, conducted a real-time survey on UK firms and showed that about 37% of employees reported working remotely in both April and May 2020. Moreover, Eurostat data, collected in the Labor Force Survey,^6^ showed that before the pandemic, in 2019 only about 5% of the EU workforce worked from home, while in 2020 this percentage more than doubled, as almost 12% of workers moved to some sort of smart-working. Figure 1 clearly shows that Italy and the EU followed the same trend and that the burst of the pandemic gave a strong push to switch to remote working (Grzegorczyk et al. 2021). Effects of the COVID-19 emergency on working hours have been examined in Fan and Moen (2022), for various categories of people working remotely during the pandemic.

Recently, Sostero et al. (2020) proposed a new index to measure “teleworkability”, meaning with this term the possibility for a job to be done remotely, based on the task contents (physical, intellectual, and social interaction tasks), the methods and tools of work. Its authors’ calculations suggested that before COVID-19, telework was not adopted at its best, as many “teleworkable jobs” were still performed in a traditional office or firm. In addition to this, the gap between teleworkability and the real usage of telework was larger for clerical support workers than for managers and professionals, pointing to what they defined as a “hierarchy effect”: before the pandemic, *“access to telework depended more on occupational hierarchy and associated privileges than the task composition of the work”* (Sostero et al. 2020). These are the reasons why pre-pandemic levels of telework were regularly minimal, while they reached their maxima during the pandemic.

To analyze the impact of COVID-19 on social soft skills we rely on a machine learning technique. In particular, we follow the seminal work Mazumder et al. (2010) on matrix completion, which refers to the task of filling in missing elements of a partially observed matrix. Matrix completion techniques have been widely applied in recommender systems (Ricci et al. 2011) to derive users’ preferences knowing the tastes of similar users and/or to suggest products that could match these preferences. Missing data is a problem that is frequently encountered by researchers in their studies, and is common in different disciplines. As Ma and Chen (2019) pointed out, in the current era of big data, it is quite likely for incomplete observations to occur. In order to deal with this issue, it is always possible to work with a balanced panel obtained by removing a subset of observations (including incomplete ones), but this is inefficient since in so doing one throws away possibly useful information from some series. This has led researchers to develop simple methods able to replace unobserved values, e.g. with zero or with the empirical mean computed on the available values, as well as more sophisticated methods, which are able to fully specify the data generation process and the missing data originating mechanism. The most classical literature on matrix completion (Candès and Recht 2009; Candès and Plan 2010; Mazumder et al. 2010) attempts to impute the missing entries of a matrix by assuming that the complete one (which is, however, only partially observed by the matrix completion algorithm) is the sum of a low-rank matrix and a random matrix representing noise and that the positions of missing entries are also random. Imposing a low-rank structure to the original unperturbed matrix—which is often assumed in modern factor-based econometric models (Fan et al. 2021) and models for time series forecasting (Gillard and Usevich 2018)—suggests the inclusion of a term depending on a regularization parameter inside the objective function of the matrix completion optimization problem. Such a regularized optimization problem is typically easier to solve when its regularization term depends on the nuclear norm of the reconstructed matrix (indeed, in such a situation, the problem is convex). This also holds in the case of complex missing data patterns (Athey et al. 2021). In Athey et al. (2021), the application of matrix completion was extended to causal inference in panel data settings, overcoming the two prevalent approaches to missing outcomes in econometrics: lagged outcomes regression (Imbens and Rubin 2015), which imputes missing potential outcomes exploiting observed outcomes for units having similar values for such outcomes in the past periods; and synthetic control (Abadie et al. 2015; Doudchenko and Imbens 2016), which attributes missing control outcomes to treated units by looking for suitable weighted empirical averages of control units matching such treated units in terms of lagged outcomes. Athey et al. (2021) proposed estimators based on matrix completion in a context in which a subset of units undergoes a treatment for a finite period of time, and the objective is to estimate counterfactual (i.e., in this case, untreated) outcomes for the various treated units/period combinations. Hence, such counterfactual values are used to predict the missing elements of a matrix, which correspond indeed to treated units/periods.

Similarly, in this work we use data on occupations and skills, treating our units with three different levels of the possible impact of COVID-19 on those working conditions related to, e.g. exposure to disease and infections, physical proximity, and working remotely, so as to get differences in the average importance levels of social soft skills (simulated versus predicted by matrix completion) for these three possible different levels of the spread of the pandemic. The matrix completion optimization problem is formulated as a nuclear-norm regularized optimization problem and is solved via a state-of-the-art algorithm, called Soft Impute (Mazumder et al. 2010). This allows us to derive our counterfactual units, making it possible to compare predictions of pre- and (simulated) post-COVID-19 skill average importance levels, by computing their differences. In other words, in our context, the counterfactual analysis stands for the assessment of the average change in the predictions generated by matrix completion as an effect of a perturbation of the matrix to which matrix completion is applied. It is worth mentioning that, unlike in Athey et al. (2021), where missing values represent missing potential outcomes to be imputed, the positions of the missing entries in the present work are artificially (and randomly) generated, and have no such interpretation. In Athey et al. (2021), one observes the actual outcomes under treatment for the treated units after treatment, and the outcomes under control for the control units both before and after treatment of the treated units. In the present work, the outcomes after COVID-19 are simulated, and missingness is not related to treatment. Another difference is that in Athey et al. (2021), missingness is dependent on time, whereas in the present study—which is more related to traditional literature on matrix completion—we base our predictions not only on the simulated changes in working conditions but also on a subset of skill average importance levels of professions in the pre-treatment phase (before the COVID-19 crisis). Moreover, positions of missing entries are randomly extracted from specific columns, as detailed later in Sect. 4.1.

## Data

### Data sources

In our work, we combine data coming from three sources: first, we make use of the Italian equivalent of the O*Net database, namely the *Survey on Occupations* (ICP, Indagine Campionaria sulle Professioni),^7^ run by the Italian National Institute for the Analysis of Public Policy (INAPP); second, we exploit the distribution of occupational employment at both the 1-digit and 2-digit level, by considering respectively 21 ATECO^8^ economic sections and, at a higher granularity level, 88 ATECO economic sectors. The two sources of these distributional data are the Microdata For Research (MFR) on the Continuous Detection of Labor Force (RCFL), provided for research purposes by the Italian National Institute of Statistics (ISTAT), and the ISTAT data on the Italian population.^9^ Similarly, we also get from these last two sources the distribution of occupational employment in 14 different age groups. Such employment-based occupation weights are then exploited to predict possible effects of the COVID-19 pandemic on the social soft-skills endowment of the different production sections/sectors and age groups.

The ICP is a survey on workers, which was run last in 2013. It encloses a sample of about 16 000 Italian workers referred to 796 occupations, following the CP2011 classification (which is the Italian equivalent of the ISCO-08 ILO’s classification).^10^ The same number of workers (20) is interviewed for each profession, as the goal is to give the same importance to each profession. The sample stratification is representative of the sector, occupation, firm size, and geography.^11^ The ICP dataset collects the answers of the sample workers with an exceptionally detailed questionnaire which includes attitudes, generalized working activities, knowledge, skills, values, working styles, and working conditions.^12^

Thanks to the large amount of information contained in the ICP dataset, we were able to focus not only on skills and competencies, but also on those related variables that account for working attitudes, conditions, and styles as well as generalized working activities. As the focus of our analysis is on social soft skills, we reclassified such items and, among those, we identified 21 items associated with social soft skills, as reported in Table 1.^13^ The identification of these 21 social soft skills stems from the consideration that in order to employ them, workers need to interact and relate with other people, otherwise it is impossible to make use of them. Then, following Barbieri et al. (2022), we also identified 5 of the available working conditions that were severely impacted by the spread of COVID-19 (see Table 2, which is described in detail in the next section).

**Table 1:** Identified (columns of the ICP matrix associated with) social soft skills and, for each of them, its original ICP section, and its ICP item code

Identified social soft skill	Original ICP section	ICP item code
Listening actively		C2A
Speaking		C4A
Monitoring		C10A
Social perception		C11A
Coordination with others		C12A
Persuading	*Competencies*	C13A
Negotiating		C14A
Teaching		C15A
Service orientation		C16A
Time management		C32A
Human resources management		C35A
Leadership		F4
Cooperating	*Working styles*	F5
Taking care of others		F6
Teamworking		F7
Coordinating		G33A
Managing working groups		G34A
Training		G35A
Guiding, directing and	*Generalized working activities*	G36A
motivating the subordinates		
Making people grow		G37A
Consultancy		G38A

The final occupation matrix considered in our analysis contains $m=796$ rows which refer to professions and $n=255$ columns, which refer to answers to questions that, according to the ICP survey design, were originally collected in macro-categories such as skills, competencies, working attitudes, working styles, generalized working activities, and working conditions. Among these columns, 21 refers to the identified social soft skills, and 5 to the identified working conditions. Each entry in position (*i*, *j*) of the occupation matrix represents the average importance level^14^ (expressed as a percentage, and averaged over the respondents) of skill/competence/working attitude/working style/generalized working activity/working condition *j* for the profession *i*.

**Fig. 2:**
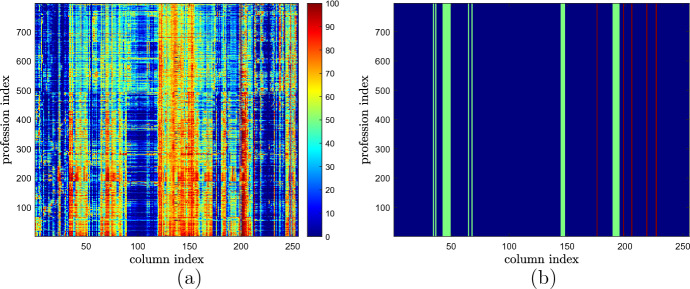
**a** Original occupation matrix. The row index refers to the professions considered in the ICP survey (numbered from 1 to 796). The column index refers to answers to the questions asked in the ICP survey (numbered from 1 to 255). The matrix entry in position (*i*, *j*) is a percentage (from 0 to 100%, depicted respectively in blue and in red), which expresses the average importance level (averaged over the respondents) of the skill/competence/working attitude/working style/generalized working activity/working condition *j* by worker type *i*. **b** Green: columns of the occupation matrix associated with the 21 selected social soft skills; red: columns of the occupation matrix associated with the 5 selected working conditions; blue: other columns. (Color figure online)

**Fig. 3:**
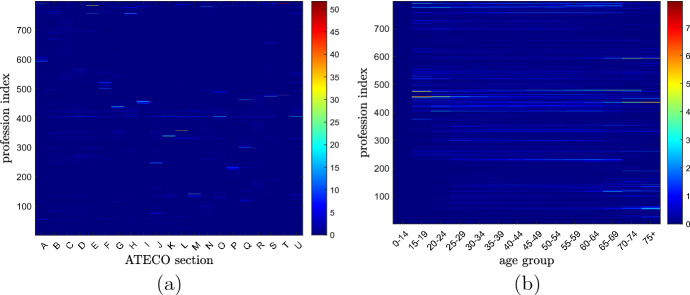
**a** Percentage of Italian workers per profession in each of the 21 ATECO sections (year: 2020). **b** Percentage of Italian workers per profession in each age group (year: 2020). (Color figure online)

The reason to keep in the dataset under study a large set of columns, not all directly related to social soft skills (i.e. the other columns of the ICP matrix, different from the 21 columns that we identified as being associated with social soft skills) is that the machine-learning technique adopted for the analysis (matrix completion) has the ability to discover automatically, if present, possible hidden associations among the columns of a matrix, with the aim of improving its prediction accuracy.^15^ The elements of the occupation matrix are visualized in Fig. 2a, whereas the locations in that matrix of the columns associated with the 21 selected social soft skills and with the 5 selected working conditions (the ones manipulated in the various simulated post-COVID-19 scenarios) are reported in Fig. 2b, respectively in green and in red. Moreover, Fig. 3a represents, for each ATECO section, the percentage of Italian workers associated with each profession in the occupation matrix (in the figure, professions are numbered from 1 to 796, in the same order as in the occupation matrix).^16^ Finally, Fig. 3b reports, for each age group, the percentage of Italian workers associated with each profession in the occupation matrix.^17^

### Data manipulation

The ICP survey contains variables that are extremely useful to illustrate the potential risks workers faced during the COVID-19 emergency and formulate hypotheses on how the labor market will evolve in the near future. In particular, for every profession, the survey directly asks workers about their physical proximity and disease exposure, relying respectively on the following questions: “During your work are you physically close to other people?” and “How often does your job expose you to diseases and infections?”. A score, which belongs to a scale from 0 to $100\%$ (i.e., from less to more intense), is computed for each job at the 5-digit level. Following Barbieri et al. (2022), we identified five working conditions that were mostly affected (negatively or positively) by the spread of the COVID-19 pandemic:

1. Working remotely (using computers for information processing);
2. Face-to-face discussions (“How often do you have to have face-to-face discussions with individuals or teams in this job?”);
3. Dealing with external customers (“How important is it in carrying out your work to interact in first person with external customers or in general with the public?”);
4. Physical proximity (“To what extent does this job require the worker to perform job tasks in close physical proximity to other people?”);
5. Exposure to disease and infections (“How often does this job require exposure to disease/infections?”).

We assumed that with the surge and the spread of the COVID-19 pandemic, workers would face higher exposure to disease and infections and higher levels of working remotely. At the same time, physical proximity would be reduced together with the possibility of having face-to-face discussions and dealing with external customers. Therefore, in our simulated matrices, all the entries in the columns related to points 1 and 5 above were increased, while all the entries in the remaining treated columns were reduced, as shown in Table 2.

**Table 2:** Working conditions mostly affected by the spread of the COVID-19 pandemic and their implied changes

ICP item code	Working condition	Change
G19A	*Working with computers*	Positive
H1	*Face-to-face discussions*	Negative
H8	*Dealing with external customers*	Negative
H21	*Physical proximity*	Negative
H29	*Exposure to disease/infections*	Positive

Since we do not exactly know how much the single working conditions above were affected by the spread of the COVID-19 pandemic (and by the related consequent countermeasures), we simulated their possible effects by considering the following post-COVID-19 scenarios:

- *Low impact: 25%* (or “COVID 25” scenario), i.e., all the entries in the columns related to the five working conditions affected by COVID-19 were reduced (increased) by 25%;
- *Medium impact: 50%* (or “COVID 50” scenario), i.e., all the entries in the columns related to the five working conditions affected by COVID-19 were reduced (increased) by 50%;
- *High impact: 75%* (or “COVID 75” scenario), i.e., all the entries in the columns related to the five working conditions affected by COVID-19 were reduced (increased) by 75%.

In constructing the matrices associated with the simulated post-COVID-19 scenarios reported above, it is worth recalling that all the elements of such matrices represent percentages, thus if any entry went above 100% due to the simulated increase, it was thresholded at the 100% level. In the analysis, we also considered a baseline scenario (“no COVID” scenario) in which the occupation matrix was not perturbed. In the following, the baseline scenario is also denoted by the superscript “$^{(0)}$”.

Assuming that it will take long for workers to get back to the traditional way of performing their job, when not impossible, the results (reported in the following sections) relative to the above simulated post-COVID-19 scenarios could be read as predictions of what would imply for professions and social soft skills a reduction of face-to-face contact and proximity, an increase smart-working, and so on. While it is true that the pandemic has been a once-in-a-lifetime experience and that some measures and precautions will be removed in the future, it is also true that the shock it caused brought about some permanent changes in society as a whole across economic sectors.

## Methodology

### Matrix completion

In our analysis, we applied Matrix Completion (MC) to estimate the difference in the average importance levels of social soft skills before and after the spread of COVID-19 in the Italian economic sectors. To apply MC to each of our occupation matrices (one for each simulated post-COVID-19 scenario and another one for the baseline one), we artificially generated several partially observed matrices from it by respectively selecting 10%, 25% and 50% of its rows randomly and obscuring all entries in the 21 columns associated with the social soft skills. We focused each time on the prediction capability of MC on every single row (occupation), from which elements of the test set were extracted (among the ones initially obscured). All the remaining obscured entries were associated with the validation set, whereas all the remaining not obscured entries were associated with the training set.^18^ In particular, for each (simulated post-COVID-19 or baseline) scenario and percentage of obscured entries in the selected columns, MC was applied for 200 different training sets (MC repetitions). Several validation/test sets were generated for each training set, by changing each time the row associated with the test set. In each of the various MC applications, the elements’ positions in the training/validation/test set were the same for all the (simulated post-COVID-19 and baseline) scenarios.

In summary, we considered the following nuclear-norm regularized MC optimization problem:

1 $$\begin{aligned} \underset{\textbf{Z} \in \mathbb {R}^{m \times n}}{\textrm{minimize}} \left( \frac{1}{2} \sum _{(i,j) \in \Omega ^{\textrm{tr}}} \left( M_{i,j}-Z_{i,j} \right) ^2 + \lambda \Vert \textbf{Z}\Vert _*\right) , \end{aligned}$$

where $\Omega ^\textrm{tr}$ is a training set of positions (*i*, *j*) corresponding to the known elements of the partially observed matrix $\textbf{M} \in \mathbb {R}^{m \times n}$, $\textbf{Z} \in \mathbb {R}^{m \times n}$ is the completed matrix,^19^$\Vert \textbf{Z}\Vert _*$ is its nuclear norm (i.e., the summation of all its singular values), and $\lambda \ge 0$ represents a regularization constant. The rank of the resulting completed matrix was determined implicitly by the regularization via the presence of the additive penalty term in the objective function. Then, we solved the optimization problem (1) by applying the Soft Impute algorithm (Mazumder et al. 2010). This is proved to converge to an optimal solution to that optimization problem. Several instances of such a problem were solved by the Soft Impute algorithm by considering different choices of the set of obscured entries (the ones that did not belong to the training set). For each instance, the best value of $\lambda$ was found by minimizing a suitable error on the validation set, whereas the final performance was evaluated on the test set. Further details on the MC optimization problem (1) and on the Soft Impute algorithm can be found in Metulini et al. (2022) and in the Supplementary Material of Gnecco, Nutarelli, and Riccaboni (2022).

**Fig. 4:**
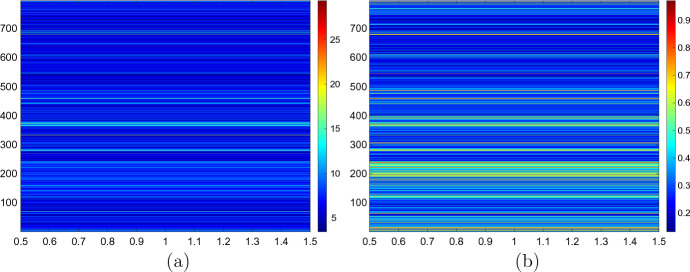
**a** Empirical mean and **b** empirical standard deviation of the Root Mean Square Error (RMSE) of prediction on the test set (one for each row of the matrix to be completed) for the “COVID 50” scenario and a specific choice ($25 \%$) for the percentage of missing entries in the columns associated with social soft skills. Each colored line illustrates graphically the empirical mean (respectively, standard deviation) of the RMSE on the whole test set located on the corresponding row. (Color figure online)

Figure 4 shows (focusing for illustrative purposes on one of the three simulated post-COVID-19 scenarios considered, i.e., the “COVID 50” scenario, and considering the case of 25% missing entries in the selected columns)^20^ that the algorithm employed exhibited a quite satisfactory prediction capability for the specific learning task, as the (empirical) mean of the Root Mean Square Error (RMSE) of MC prediction (on the test set) per profession^21^ turned out to be typically smaller than 15%. Moreover, its (empirical) standard deviation per profession turned out to be much smaller (its maximum value turned out to be around 0.94%).

**Fig. 5:**
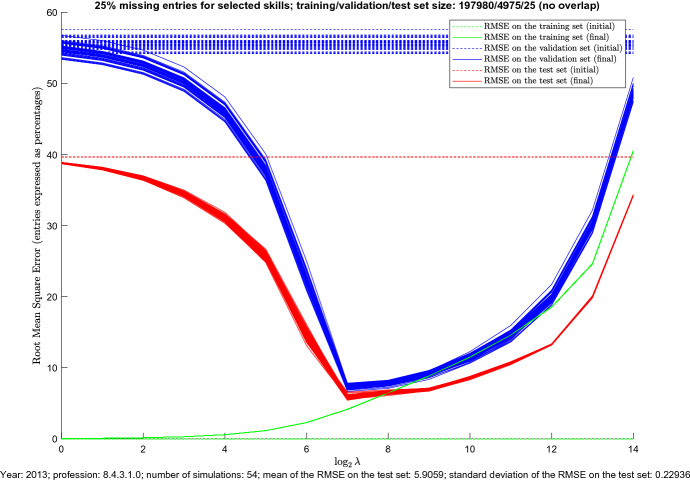
RMSEs of prediction on the training, validation and test sets as functions of the regularization parameter $\lambda$, for all the repetitions associated with the same test set related to a specific profession, represented by its associated code at the 5-digit level (“COVID 50” scenario, $25\%$ of missing entries in the selected columns). (Color figure online)

The RMSE of the MC prediction, as evaluated on the validation and test sets, was typically decreasing with respect to the regularization parameter $\lambda$ up to its minimum value, as shown in Fig. 5 for a specific profession (chosen for illustrative purposes) in the case of the same post-COVID-19 scenario and the same percentage of missing entries as in Fig. 4. A similar behavior was obtained on the test set, as the figure illustrates.^22^ The variability of the curves due to changing the training and validation sets (fixing the test set related to a specific profession) turned out to be quite small (see Fig. 5). So, MC showed a high generalization capability in this specific application. It is worth mentioning that the baseline scenario was also studied in Gnecco, Landi, and Riccaboni (2022), focusing, however, on a different subset of soft skills to evaluate the MC performance. In that work, possibly due to the absence of any perturbation on the original occupation matrix, the MC application to that scenario produced even smaller (empirical) means and standard deviations for the RMSE (on the test set) per profession achieved by the MC prediction.

### Measures of the simulated COVID-19 impact on social soft-skills endowment

In the remaining of this work, we use the following notation: *J* is the set of 21 social soft skills identified, $j \in J$ denotes one of them, $l \in L=\{10\%, 25\%, 50\%\}$ denotes one of the three considered percentages of missing entries in the selected columns, $r \in \{1, \ldots , r_i\}$ refers to one of the $r_i$ MC repetitions for which elements of row *i* were in the test set, whereas, for each simulated post-COVID-19 scenario, $predicted^{\,i,j,l,r}_{social\,soft\,skills}$ is the MC prediction associated with a specific choice of *i*,*j*,*l*, and *r*, whereas $predicted^{\,i,j,l,r;(0)}_{social\,soft\,skills}$ is the corresponding MC prediction in the baseline scenario. For simplicity of notation, the dependence of $predicted^{\,i,j,l,r}_{social\,soft\,skills}$ from each simulated post-COVID-19 scenario is not indicated explicitly.

As the focus of our analysis consists in investigating the simulated COVID-19 impact on social soft-skills endowment (i.e., in assessing how much the MC predictions on social soft skills changed on average when moving from the baseline scenario to each of the other simulated post-COVID-19 scenarios), we started by defining, for each of the three simulated post-COVID-19 scenarios, and for each profession *i*, the following quantity

2 $$\begin{aligned} \Delta predicted^{\,i,j,l,r}_{social\,soft \,skills}:=predicted^{\,i,j,l,r}_{social\,soft\,skills}-predicted^{\,i,j,l,r;(0)}_{social\,soft\,skills}, \end{aligned}$$

i.e. the difference between the MC prediction (in the repetition *r*) for the test set element of the occupation matrix in position (*i*, *j*) for the simulated post-COVID-19 scenario considered and the selected percentage *l* of missing entries in the selected columns, and the MC prediction for an element in the same position, but referring to the baseline scenario. In other words, for each simulated post-COVID-19 scenario, by manipulating the elements belonging to the columns of the original ICP matrix related to the five selected working conditions, we quantified, for each profession, *i*, each of the 21 columns *j* related to social soft skills, each of the three considered percentages *l* of obscured entries in the selected columns, and each repetition *r*, how much the predictions obtained by MC on the elements belonging to each of the 21 columns related to social soft skills changed with respect to the baseline scenario when they were in the test set.

Starting from Eq. (2), we defined additional quantities, which were considered in various analyses, whose results are reported later in Sect. 5. These additional quantities are presented and discussed in the following list.

1. Interpreting the original quantities $\Delta predicted^{\,i,j,l,r}_{social\,soft\,skills}$ obtained by varying *r* in Eq. (2) as identically distributed realizations of a random variable $\Delta predicted^{\,i,j,l}_{social\,soft\,skills}$, we computed its empirical mean and empirical standard deviation, respectively, according to the two following expressions:
  3 $$\begin{aligned}{} & {} \overline{\Delta predicted}^{\,i,j,l}_{social\,soft\,skills}=\frac{1}{r_i} \sum _{r=1}^{r_i} \Delta predicted^{\,i,j,l,r}_{social\,soft\,skills}, \end{aligned}$$
  4 $$\begin{aligned}{} & {} \hat{\sigma }_{\Delta predicted^{\,i,j,l}_{social\,soft\,skills}}=\sqrt{\frac{1}{r_i-1} \sum _{r=1}^{r_i} \left( \Delta predicted^{\,i,j,l,r}_{social\,soft \,skills}-\overline{\Delta predicted}^{\,i,j,l}_{social\,soft\,skills}\right) ^2}. \end{aligned}$$
  It is worth remarking that, if for a specific test set (associated with a particular profession *i*), the optimal choice of the regularization parameter $\lambda$ did not depend on the validation set, then the quantities $\Delta predicted^{\,i,j,l,r}_{social\,soft\,skills}$ would be also independent realizations of the random variable $\Delta predicted^{\,i,j,l}_{social\,soft\,skills}$, and the (empirical) Standard Error (SE) of the estimate $\overline{\Delta predicted}^{\,i,j,l}_{social\,soft\,skills}$ (i.e., the standard deviation of that empirical mean) would be approximately equal to
  5 $$\begin{aligned} SE_{\overline{\Delta predicted}^{\,i,j,l}_{social\,soft\,skills}}=\frac{1}{\sqrt{r_i}}\hat{\sigma }_{\Delta predicted^{\,i,j,l}_{social\,soft\,skills}}. \end{aligned}$$
  In practice, as shown in Fig. 5 for a representative choice of the profession *i* associated with the test set, it turned out that the optimal choice of $\lambda$ depended very negligibly on the validation set, so it was still possible to use Eq. (5) also in this case.
2. Differently from item 1, it was not possible to assume independence of the quantities ${\Delta predicted}^{\,i,j_1,l}_{social\,soft\,skills}$ and ${\Delta predicted}^{\,i,j_2,l}_{social\,soft\,skills}$ associated with any two different choices $j_1$ and $j_2$ for *j*, due to the construction of the test set associated with each specific profession *i* (indeed, that test set is made simultaneously by all the entries in position (*i*, *j*), with $j \in J$). However, in this case, it was still possible to apply the same reasoning reported in item 1, obtaining that the random variable
  6 $$\begin{aligned} {\Delta predicted}^{\,i,l}_{social\,soft\,skills}=\frac{1}{|J|}\sum _{j \in J} {\Delta predicted}^{\,i,j,l}_{social\,soft\,skills} \end{aligned}$$
  has empirical mean
  7 $$\begin{aligned} {\overline{\Delta predicted}}^{\,i,l}_{social\,soft\,skills}=\frac{1}{|J|}\sum _{j \in J} {\overline{\Delta predicted}}^{\,i,j,l}_{social\,soft\,skills}, \end{aligned}$$
  and the standard error of the estimate ${\overline{\Delta predicted}}^{\,i,l}_{social\,soft\,skills}$ is approximately equal to
  8 $$\begin{aligned} SE_{\overline{\Delta predicted}^{\,i,l}_{social\,soft\,skills}}=\frac{1}{\sqrt{r_i}}\hat{\sigma }_{\Delta predicted^{\,i,l}_{social\,soft\,skills}}, \end{aligned}$$
  being
  9 $$\begin{aligned}{} & {} \hat{\sigma }_{\Delta predicted^{\,i,l}_{social\,soft\,skills}} \end{aligned}$$
  10 $$\begin{aligned}= & {} \sqrt{\frac{1}{r_i-1} \sum _{r=1}^{r_i} \left( \frac{1}{|J|}\sum _{j \in J} \Delta predicted^{\,i,j,l,r}_{social\,soft \,skills}-\overline{\Delta predicted}^{\,i,l}_{social\,soft\,skills}\right) ^2}. \end{aligned}$$
3. For each profession *i*, we considered the empirical mean of the average of the quantities $\Delta predicted^{\,i,j,l}_{social\,soft \,skills}$ (averaging with respect to *j* and *l*).^23^ The obtained expression for the empirical mean of the resulting random variable, denoted as ${\Delta predicted}^{\,i}_{social\,soft\,skills}$, is reported in the following equation:
  11 $$\begin{aligned} \overline{\Delta predicted}^{\,i}_{social\,soft\,skills}=\frac{1}{|L|} \sum _{l \in L} \overline{\Delta predicted}^{\,i,l}_{social\,soft\,skills}. \end{aligned}$$
  We say that for a specific profession *i*, a simulated post-COVID-19 scenario yielded a deficit in social soft-skills endowment when its empirical mean MC prediction was smaller than in the baseline scenario (i.e. when $\overline{\Delta predicted}^{\,i}_{social\,soft \,skills} < 0$). Similarly, we say that it induced a surplus in social soft-skills endowment when its empirical mean MC prediction was larger than the one in the baseline scenario (i.e. when $\overline{\Delta predicted}^{\,i}_{social\,soft\,skills} > 0$). Based on item 2, given the independence of the results obtained for different values of the percentage *l* of obscured entries in the selected columns, it was possible to approximate the standard error of the estimate $\overline{\Delta predicted}^{\,i}_{social\,soft\,skills}$ with
  12 $$\begin{aligned} SE_{\overline{\Delta predicted}^{\,i}_{social\,soft\,skills}}=\frac{1}{|L|}\sqrt{\sum _{l \in L} (SE_{\overline{\Delta predicted}^{\,i,l}_{social\,soft\,skills}})^2}. \end{aligned}$$
4. We further aggregated the results at the level of each ATECO section, ATECO sector, age group, or simply at the level of the whole Italian working population (in the following, each specific case is clear from the context, and is presented later in Sect. 5). In more detail, we attributed a non-negative weight $w_i$ to each profession (which depended on the choice—denoted in the following by *I*—of the ATECO section, ATECO sector, age group, or of the whole Italian working population), in such a way that $\sum _{i \in I} w_i=1$. Each of these weights $w_i$ is equal to the estimated fraction of the Italian working population in 2020 either without restrictions (i.e. the whole Italian working population) or restricted to the specific ATECO section, ATECO sector, or age group *I*, whose profession is *i*. Such weights are proportional (via the factor 1/100) to the percentages of Italian workers estimated at the end of Sect. 3.1. Finally, we computed quantities that are similar to those defined in Eqs. (11) and (12), in which the missing indices are the ones that were averaged out. For instance, the following expression
  13 $$\begin{aligned} \overline{\Delta predicted}_{social\,soft\,skills}=\sum _{i \in I} w_i \overline{\Delta predicted}^{\,i}_{social\,soft\,skills} \end{aligned}$$
  refers to the empirical mean of the weighted average of ${\Delta predicted}^{\,i,j,l}_{social\,soft\,skills}$ with respect to *i*, *j*, and *l* (giving the weights $w_i$ to *i*, 1/|*J*| to *j*, and 1/|*L*| to *l*), denoted as ${\Delta predicted}_{social\,soft\,skills}$, whereas the standard error of the estimate $\overline{\Delta predicted}_{social\,soft\,skills}$ is approximately equal to
  14 $$\begin{aligned} SE_{\overline{\Delta predicted}_{social\,soft\,skills}}=\sqrt{w_i^2 \left( {SE_{\overline{\Delta predicted}^{\,i}_{social\,soft\,skills}}}\right) ^2}. \end{aligned}$$
  Similarly, the following expression
  15 $$\begin{aligned} \overline{\Delta predicted}^{\,j}_{social\,soft\,skills}=\frac{1}{|L|} \sum _{i \in I} \sum _{l \in L} w_i \overline{\Delta predicted}^{\,i,j,l}_{social\,soft\,skills} \end{aligned}$$
  refers to the empirical mean of the weighted average of ${\Delta predicted}^{\,i,j,l}_{social\,soft\,skills}$ with respect to *i* and *l* (giving the weights $w_i$ to *i* and 1/|*L*| to *l*), denoted as ${\Delta predicted}^{\,j}_{social\,soft\,skills}$, whereas the standard error of the estimate $\overline{\Delta predicted}^{\,j}_{social\,soft\,skills}$ is approximately equal to
  16 $$\begin{aligned} SE_{\overline{\Delta predicted}^{\,j}_{social\,soft\,skills}}=\frac{1}{|L|} \sqrt{ \sum _{i \in I} \sum _{l \in L} w_i^2 (SE_{\overline{\Delta predicted}^{\,i,j,l}_{social\,soft\,skills}})^2}. \end{aligned}$$

It is worth remarking that the definitions of deficits and surpluses (induced by a specific simulated post-COVID-19 scenario) for a social soft skill *j* are similar to the ones already introduced for an occupation *i*, as being obtained by replacing the sign of $\overline{\Delta predicted}^{\,i}_{social\,soft\,skills}$ with the one of $\overline{\Delta predicted}^{\,j}_{social\,soft\,skills}$. A similar comment holds for the case of $\overline{\Delta predicted}_{social\,soft\,skills}$.

Finally, to evaluate the statistical significance of the results, we adopted a Gaussian approximation^24^ for random variables like ${\Delta predicted}^{\,i}_{social\,soft\,skills}$, ${\Delta predicted}^{\,j}_{social\,soft\,skills}$, and ${\Delta predicted}_{social\,soft\,skills}$, and assumed, e.g., that an empirical mean like $\overline{\Delta predicted}^{\,j}_{social\,soft\,skills}$ was statistically different from 0 at the 95% confidence level when

17 $$\begin{aligned} 0&\notin&\bigg (\overline{\Delta predicted}^{\,j}_{social\,soft\,skills} - 1.96\, SE_{\overline{\Delta predicted}^{\,j}_{social\,soft\,skills}}. \nonumber \\{} & {} \,\,\,\,\,\,\overline{\Delta predicted}^{\,j}_{social\,soft\,skills} + 1.96\, SE_{\overline{\Delta predicted}^{\,j}_{social\,soft\,skills}}\bigg ), \end{aligned}$$

or equivalently, when $|\overline{\Delta predicted}^{\,j}_{social\,soft\,skills}| > 1.96 \, SE_{\overline{\Delta predicted}^{\,j}_{social\,soft\,skills}}$ (i.e., we applied a two-tailed *z*-test with 0 mean in the null hypothesis, standard deviation assumed to be equal to the empirical estimate $SE_{\overline{\Delta predicted}^{\,j}_{social\,soft\,skills}}$. and significance level $\alpha =0.05$). In the tables of the next Sects. 5 and 6, statistically significant results obtained according to such a test are reported with an asterisk.

## Results

In this section, we report results related to the measures of the simulated COVID-19 impact on social soft-skills endowment, which were introduced in Sect. 4.2.

In our first analysis, by varying the social soft skill $j \in J$, we evaluated the quantity $\overline{\Delta predicted}^{\,j}_{social\,soft\,skills}$—see Eq. (15)—using a weight $w_i$ for each profession *i* equal to the associated estimated fraction of the Italian working population having that occupation in 2020. The results are reported in Table 3. The table shows that statistically significant results (according to the specific statistical test performed, see Sect. 4.2)^25^ were obtained for all the cases reported. Moreover, negative empirical mean variations of the MC prediction were obtained for all the selected social soft skills, apart from consultancy (G38A). For the latter, a positive empirical mean variation of the MC prediction was obtained. It is worth observing that, for each social soft skill, the magnitude of $\overline{\Delta predicted}^{\,j}_{social\,soft\,skills}$ increased when moving from the “COVID 25” scenario to the “COVID 50” scenario, then to the “COVID 75” scenario.

Then, Table 4 reports the top 5 highest and lowest quantities $\overline{\Delta predicted}_{social\,soft\,skills}^{\,j}$ (where, for a better synthesis of the results and for easier comparison with the ones reported later in Sect. 6, the empirical means were performed also with respect to the three simulated post-COVID-19 scenarios), and the list of the corresponding social soft skills. Also in this case, statistically significant results were obtained for all the cases reported in the table.^26^ Again, a positive empirical mean variation of the MC prediction was obtained only for consultancy (G38A). Moreover, it is worth remarking that, having the empirical means been performed with respect to the three simulated post-COVID-19 scenarios, the empirical mean variations reported in Table 4 turned out to be of the same order of magnitude as the ones reported in Table 3 for the intermediate “COVID 50” scenario. It is also worth discussing the importance of most of the social soft skills highlighted in Table 4 by reporting the following considerations. During the pandemic, the ability to adapt to the new normality of working from home, lockdown, and social distancing required workers a certain capacity for coordinating with others, working under unexpected deadlines, and setting priorities. Time management, in fact, became crucial, since when working from home it is important to be able to adjust working hours to family needs. Together with these, leadership qualities are required as they reflect in social soft skills such as human resources management, listening actively, teaching, and guiding, directing and motivating subordinates. Lastly, support among colleagues as well as coordination and empathy are fundamental for teamworking.

**Table 3:** Empirical mean variation of the MC prediction for each social soft skill, and for each of the three simulated post-COVID-19 scenarios. A weighted average across all the professions, taking into account the Italian working population, has been taken. Negative values imply a deficit of social soft-skills endowment associated with the corresponding simulated post-COVID-19 scenario. In parenthesis: empirical standard errors

Social soft skill	COVID-19 impact: $\overline{\Delta predicted}^{\,j}_{social\,soft\,skills}$ ($SE_{{\overline{\Delta predicted}}^{\,j}_{social\,soft\,skills}}$)		
	25%	50%	75%
C2A *(Listening actively)*	$-$0.0131$^*$	$-$0.0355$^*$	$-$0.0481$^*$
	(0.0021)	(0.0021)	(0.0021)
C4A *(Speaking)*	$-$0.0294$^*$	$-$0.0580$^*$	$-$0.0758$^*$
	(0.0019)	(0.0019)	(0.0019)
C10A *(Monitoring)*	$-$0.0371$^*$	$-$0.0614$^*$	$-$0.0832$^*$
	(0.0024)	(0.0024)	(0.0024)
C11A *(Social perception)*	$-$0.0376$^*$	$-$0.0625$^*$	$-$0.0802$^*$
	(0.0030)	(0.0030)	(0.0030)
C12A *(Coordination with others)*	$-$0.0490$^*$	$-$0.0753$^*$	$-$0.0940$^*$
	(0.0023)	(0.0023)	(0.0023)
C13A *(Persuading)*	$-$0.0254$^*$	$-$0.0573$^*$	$-$0.0763$^*$
	(0.0031)	(0.0031)	(0.0031)
C14A *(Negotiating)*	$-$0.0160$^*$	$-$0.0369$^*$	$-$0.0492$^*$
	(0.0027)	(0.0027)	(0.0027)
C15A *(Teaching)*	$-$0.0505$^*$	$-$0.0855$^*$	$-$0.1108$^*$
	(0.0030)	(0.0030)	(0.0030)
C16A *(Service orientation)*	$-$0.0234$^*$	$-$0.0509$^*$	$-$0.0642$^*$
	(0.0030)	(0.0030)	(0.0030)
C32A *(Time management)*	$-$0.0151$^*$	$-$0.0279$^*$	$-$0.0378$^*$
	(0.0026)	(0.0026)	(0.0026)
C35A *(Human resources management)*	$-$0.0206$^*$	$-$0.0241$^*$	$-$0.0322$^*$
	(0.0026)	(0.0026)	(0.0026)
F4 *(Leadership)*	$-$0.0388$^*$	$-$0.0577$^*$	$-$0.0721$^*$
	(0.0025)	(0.0025)	(0.0025)
F5 *(Cooperating)*	$-$0.0443$^*$	$-$0.0641$^*$	$-$0.0826$^*$
	(0.0021)	(0.0021)	(0.0021)
F6 *(Taking care of others)*	$-$0.0360$^*$	$-$0.0490$^*$	$-$0.0640$^*$
	(0.0029)	(0.0029)	(0.0029)
F7 *(Teamworking)*	$-$0.0547$^*$	$-$0.0796$^*$	$-$0.0999$^*$
	(0.0029)	(0.0029)	(0.0029)
G33A *(Coordinating)*	$-$0.0411$^*$	$-$0.0645$^*$	$-$0.0829$^*$
	(0.0025)	(0.0025)	(0.0025)
G34A *(Managing working groups)*	$-$0.0434$^*$	$-$0.0690$^*$	$-$0.0909$^*$
	(0.0026)	(0.0026)	(0.0026)
G35A *(Training)*	$-$0.0231$^*$	$-$0.0518$^*$	$-$0.0704$^*$
	(0.0028)	(0.0028)	(0.0028)
G36A *(Guiding, directing and*	$-$0.0173$^*$	$-$0.0251$^*$	$-$0.0306$^*$
*motivating the subordinates)*	(0.0022)	(0.0022)	(0.0022)
G37A *(Making people grow)*	$-$0.0385$^*$	$-$0.0591$^*$	$-$0.0780$^*$
	(0.0027)	(0.0027)	(0.0027)
G38A *(Consultancy)*	0.0171$^*$	0.0245$^*$	0.0359$^*$
	(0.0029)	(0.0029)	(0.0029)

$^*$Statistically different from 0 at the 95% confidence level

**Table 4:** Social soft skills associated with the top five highest and lowest empirical mean variations—averaged over the Italian working population and also over the three simulated post-COVID-19 scenarios—of the MC prediction for each social soft skill $j \in J$. Negative values imply a deficit of social soft-skills endowment associated with the corresponding simulated post-COVID-19 scenario. In parenthesis: empirical standard errors

	COVID-19 impact: Social soft skill $\overline{\Delta predicted}^{\,j}_{social\,soft\,skills}$
	($$SE_{\overline{\Delta predicted}^{\,j}_{social\,soft\,skills}}$$)
G38A *(Consultancy)*	0.0258$^*$ (0.0016)
G36A *(Guiding, directing and motivating the subordinates)*	$-$0.0243$^*$ (0.0013)
C35A *(Human resources management)*	$-$0.0256$^*$ (0.0015)
C32A *(Time management)*	$-$0.0269$^*$ (0.0015)
C2A *(Listening actively)*	$-$0.0322$^*$ (0.0012)
C15A *(Teaching)*	$-$0.0823$^*$ (0.0017)
F7 *(Teamworking)*	$-$0.0781$^*$ (0.0017)
C12A *(Coordination with others)*	$-$0.0728$^*$ (0.0013)
G34A *(Managing working groups)*	$-$0.0678$^*$ (0.0015)
F5 *(Cooperating)*	$-$0.0637$^*$ (0.0012)

$^*$Statistically different from 0 at the 95% confidence level

In our subsequent analysis, occupations at the 5-digit level were grouped at the 1-digit level according to the ATECO classification, i.e., into ATECO economic sections, where one can find only the general characteristics of the goods and services produced: as detailed in Sect. 4.2, to each occupation *i*, we assigned a weight equal $w_i$ to its estimated fraction of workers in each ATECO section. We noticed that the empirical means $\overline{\Delta predicted}_{social\,soft\,skills}$, computed at the level of each ATECO section, turned out to be concentrated in a small interval, approximately $[-0.22,0.11]$ (see Table 5). Sign and magnitude turned out to be typically consistent in the three simulated post-COVID-19 scenarios considered (see again Table 5). For this analysis, the results reported in the table turned out to be typically (but not always) statistically significant. Limiting the discussion to the results that turned out to be statistically significant at least for one simulated post-COVID-19 scenario, the empirical mean variations of the MC prediction were typically negative, with a few exceptions related to ATECO sections for which they were positive, i.e, in the case of the following ATECO sections (5 over 21): agriculture, forestry and fisheries (A); mining and minerals from quarries and mines (B); water supply, sewerage, waste management and remediation (E); transportation and storage (H); rental, travel agencies, business support services (N).

In detail, Table 6 reports MC results obtained for the five highest and lowest $\overline{\Delta predicted}_{social\,soft\,skills}$, when the weighted averages were computed this time by aggregating the professions at the 2-digit level according to the ATECO classification, i.e., into ATECO sectors. For this analysis, the results reported in the table turned out to be always statistically significant.^27^ The ATECO sector with the highest empirical mean variation of the MC prediction was surveillance and investigation services (N/80), whereas the ATECO sector with the lowest empirical mean variation of the MC prediction was retail trade (excluding that of motor vehicles and motorcycles) (G/47). It is also worth noting that the empirical mean variations of the MC prediction reported in Table 6 (which refer to a subset of ATECO sectors) turned out to have a larger order of magnitude with respect to the empirical mean variations of the MC prediction reported in Table 5 (which refer to all the ATECO sections). This may depend on the fact that these two analyses refer to two different levels of aggregation, and that Table 6 reports only the 5 highest and lowest empirical mean variations among all the 88 ATECO sectors, whereas Table 5 reports the empirical mean variations for all the 21 ATECO sections. Finally, the results illustrated in Tables 5 and 6 are consistent in the sense that the ATECO sections associated with the ATECO sectors reported in Table 6 had typically the same sign of the empirical mean variation of the MC prediction as the related ATECO sectors.^28^

**Fig. 6:**
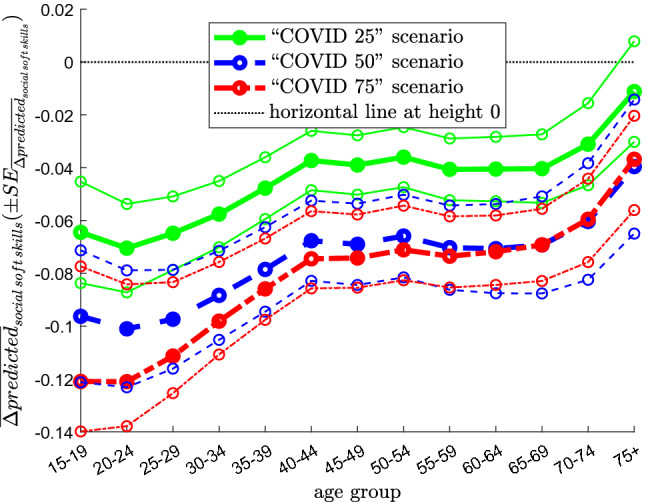
Empirical mean variation of the MC prediction ($\overline{\Delta predicted}_{social\,soft\,skills}$) as a function of the age group (excluding the age group 0–14), for each of the three simulated post-COVID-19 scenarios. A weighted average across all the professions within each age group was taken. Negative values imply a deficit of social soft-skills endowment associated with the corresponding simulated post-COVID-19 scenario. The figure also represents, for each scenario, the band centered on the empirical mean variation of the MC predictions, with half-bandwidth equal to 1.96 empirical standard errors. The horizontal line at height 0 is reported, too, for checks of statistical significance. (Color figure online)

Additionally, Fig. 6 reports the empirical means $\overline{\Delta predicted}_{social\,soft\,skills}$ computed within each age group (excluding the age group 0–14, for which there are no workers). As highlighted by the dashed lines reported in the figure, the results turned out to be statistically significant in all the analyses performed, apart from the one made for the age group $75+$ in the case of the “COVID 25” scenario. The figure shows that the lowest empirical mean variations (but the highest ones in absolute value) were obtained in correspondence with the four youngest working age groups, i.e., 15–19, 20–24, 25–29, and 30–34. Notably, the empirically estimated standard errors reported in Tables 5 and 6 and in Fig. 6 turned out to be larger than the ones reported in Tables 3 and 4, respectively. A possible explanation for this is that, in the case of Tables 3 and 4, each weighted average was performed with respect to the whole set of the 796 professions.

**Table 5:** Empirical mean variation of the MC prediction for each ATECO section and for each of the three simulated post COVID-19 scenarios. A weighted average across all the professions of each ATECO section was taken. Negative values imply a deficit of social soft-skills endowment associated with the corresponding simulated post-COVID 19 scenario. In parenthesis: empirical standard errors

ATECO section	COVID-19 impact: $\overline{\Delta predicted}_{social\,soft\,skills}$ ($SE_{\overline{\Delta predicted}_{social\,soft\,skills}}$)		
	25%	50%	75%
A *(Agriculture, forestry and fisheries)*	0.0988$^*$	0.0898$^*$	0.1134$^*$
	(0.00180)	(0.0238)	(0.0197)
B *(Mining and minerals from quarries and mines)*	0.0335$^*$	0.0175	0.0397$^*$
	(0.0107)	(0.0136)	(0.0109)
C *(Manufacturing activities)*	$-$0.0133$^*$	$-$0.0374$^*$	$-$0.0353$^*$
	(0.0058)	(0.0075)	(0.0053)
D *(Supply of electricity, gas, steam and air conditioning)*	$-$0.0159	$-$0.0461$^*$	$-$0.0532$^*$
	(0.0110)	(0.0131)	(0.098)
E *(Water supply, sewerage, waste management and*	0.0629$^*$	0.0544$^*$	0.1056$^*$
*remediation)*	(0.0184)	(0.0028)	(0.0206)
F *(Construction)*	$-$0.0241	$-$0.0433$^*$	$-$0.0583$^*$
	(0.0141)	(0.0210)	(0.0159)
G *(Wholesale and retail trade)*	$-$0.1528$^*$	$-$0.1865$^*$	$-$0.2225$^*$
	(0.0195)	(0.0260)	(0.0196)
H *(Transportation and storage)*	0.0719$^*$	0.0557$^*$	0.0591$^*$
	(0.0174)	(0.0200)	(0.0178)
I *(Accomodation and catering services)*	$-$0.1228$^*$	$-$0.1595$^*$	$-$0.1899$^*$
	(0.0178)	(0.0247)	(0.0178)
J *(Information and communication services)*	$-$0.0447$^*$	$-$0.0700$^*$	$-$0.0764$^*$
	(0.0143)	(0.0153)	(0.0117)
K *(Financial and insurance activities)*	$-$0.0851$^*$	$-$0.1090$^*$	$-$0.0998$^*$
	(0.0211)	(0.0288)	(0.0184)
L *(Real estate activities)*	$-$0.0013	$-$0.0407$^*$	$-$0.0964$^*$
	(0.0319)	(0.0365)	(0.0260)
M *(Professional, scientific, and technical activities)*	0.0223	$-$0.0039	0.0031
	(0.0267)	(0.0317)	(0.0249)
N *(Rental, travel agencies, business support services)*	0.0272$^*$	0.0029	0.0209
	(0.0117)	(0.0191)	(0.0115)
O *(Public administration and defense; social security)*	$-$0.0433$^*$	$-$0.0693$^*$	$-$0.0595$^*$
	(0.0132)	(0.0163)	(0.0137)
P *(Education)*	$-$0.0905$^*$	$-$0.1569$^*$	$-$0.1799$^*$
	(0.0231)	(0.0334)	(0.0243)
Q *(Healthcare and social services)*	$-$0.0558$^*$	$-$0.1051$^*$	$-$0.1194$^*$
	(0.0149)	(0.0255)	(0.0165)
R *(Art, sport and entertainment)*	$-$0.0267$^*$	$-$0.0660$^*$	$-$0.0731$^*$
	(0.0126)	(0.0188)	(0.0137)
S *(Other service activities)*	$-$0.1292$^*$	$-$0.1556$^*$	$-$0.1630$^*$
	(0.0212)	(0.0283)	(0.0207)
T *(Activities of households as employers of domestic*	$-$0.0410$^*$	$-$0.0513$^*$	$-$0.0241
*personnel)*	(0.0424)	(0.0564)	(0.0374)
U *(Extraterritorial organizations and bodies)*	0.0056	$-$0.0226	$-$0.0167
	(0.0133)	(0.0157)	(0.0128)

$^*$Statistically different from 0 at the 95% confidence level

**Table 6:** ATECO sectors (and corresponding previous level ATECO sections) associated with the top 5 highest and lowest empirical mean variations of the MC prediction within each ATECO sector, also averaged over the three simulated post-COVID-19 scenarios. A weighted average across all the professions of each ATECO sector was taken. Negative values imply a deficit of social soft-skills endowment associated with the corresponding simulated post-COVID-19 scenario. In parenthesis: empirical standard errors

	COVID-19 impact: ATECO section/sector $\overline{\Delta predicted}_{social\,soft\,skills}$ ($SE_{\overline{\Delta predicted}_{social\,soft\,skills}}$)
N/80 *(Surveillance and investigation services)*	0.2304$^*$ (0.0354)
N/81 *(Service activities for buildings and landscape)*	0.1149$^*$ (0.0259)
A/1 *(Agricultural crops and production of animal products,*	0.1145$^*$ (0.0125)
*hunting and related services)*	
E/38 *(Activities of collection, processing and waste disposal;*	0.1125$^*$ (0.0160)
*materials recovery)*	
H/49 *(Land transport and transport by ducts)*	0.0954$^*$ (0.0182)
G/47 *(Retail trade (excluding that of motor vehicles and*	$-$0.2468$^*$ (0.0188)
*motorcycles))*	
S/96 *(Other personal service activities)*	$-$0.2112$^*$ (0.0201)
N/79 *(Activities of the services of the travel agencies, of the tour*	$-$0.2099$^*$ (0.0156)
*operators and booking services and related activities)*	
I/56 *(Restaurant services activities)*	$-$0.1827$^*$ (0.0133)
Q/88 *(Non-residential social assistance)*	$-$0.1579$^*$ (0.0151)

$^*$Statistically different from 0 at the 95% confidence level

**Fig. 7:**
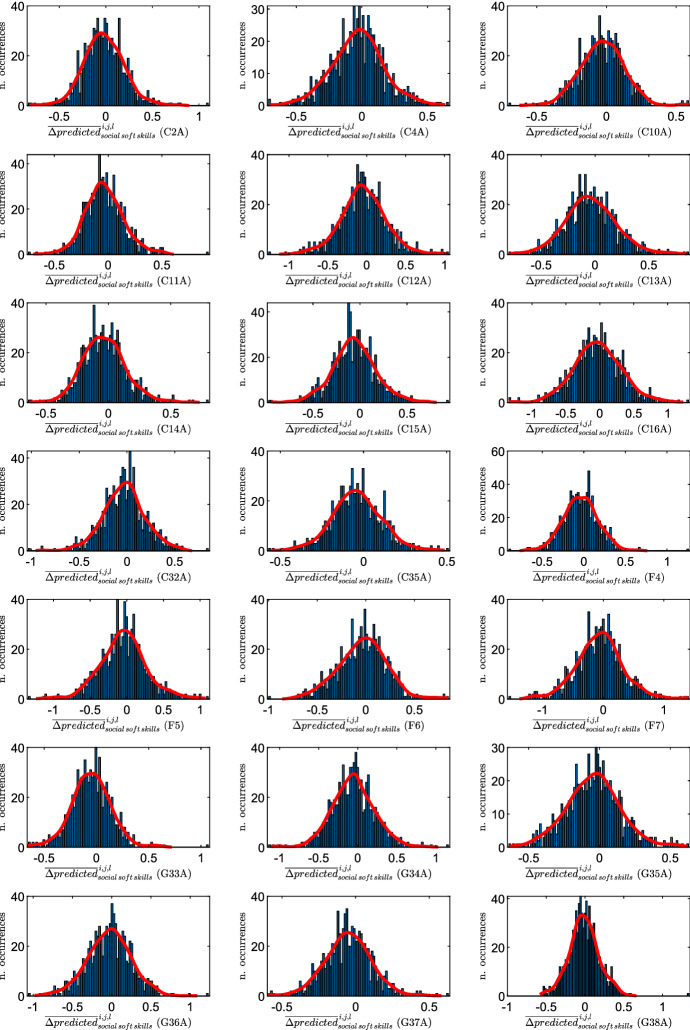
Histograms of the quantities $\overline{\Delta predicted}^{\,i,j,l}_{social\,soft\,skills}$ for the various social soft skills *j*, $l=25\%$, and the “COVID 50” scenario. In each histogram, *j* and *l* are fixed, whereas *i* varies. (Color figure online)

Finally, Fig. 7 reports, as an example for the “COVID 50” scenario and the case of 25% missing entries in the selected columns (i.e., the case $l=25\%$), the histograms of the quantities $\overline{\Delta predicted}^{\,i,j,l}_{social\,soft\,skills}$ for each social soft skill $j \in J$ (in each histogram, *j* and *l* are fixed, whereas *i* varies). The various plots reported in Fig. 7 complete this part of our analysis showing that the social soft skills for which the largest dispersions of the $\overline{\Delta predicted}^{\,i,j,l}_{social\,soft\,skills}$ around the respective means (with respect of professions) were obtained are coordination with others (C12A), service orientation (C16A), cooperating (F5), teamworking (F7), managing working groups (G34A), and guiding, directing and motivating the subordinates (G36A).

Concluding, it follows from the results reported in this section that, in the set of social soft skills, cooperating, managing working groups, coordination with others, teamworking, and teaching turned out to be among the most negatively impacted social soft skills in the simulated post-COVID-19 scenarios (i.e., the ones experiencing the most negative decreases of MC predictions of average importance levels of social soft skills), whereas a positive impact was obtained only for consultancy. Moreover, ATECO sections related to commercial activities, tourism, and education turned out to be among the most negatively impacted ones in the simulated post-COVID-19 scenarios, whereas the most negatively impacted age groups turned out to refer to workers under 35 years old.

## Robustness checks

After having considered in Table 4 of Sect. 5 the aggregated effect on the 21 selected social soft skills of the variations of all the 5 selected working conditions, we repeated the analysis by modifying each time only the column of the original occupational matrix that is associated with one among those working conditions. In order to limit the computational effort, we reduced the number of MC repetitions to 20 (instead of 200). This was also motivated by the low standard deviations of the MC results, already illustrated in Fig. 5.

Tables 7 and 8 show the obtained results. Every section of each of the two tables refers to one of the five altered working conditions and reports the three social soft skills *j* associated with the highest (lowest) variation $\overline{\Delta predicted}^{\,j}_{social\,soft\,skills}$ obtained as a consequence of the alteration of that working condition (again, the empirical means were performed also with respect to the three simulated post-COVID-19 scenarios).

**Table 7:** Three social soft skills with the highest empirical mean variation — averaged over the Italian working population and also over the three simulated post-COVID-19 scenarios — of the MC prediction for every single altered condition. Negative values imply a deficit of social soft-skills endowment associated with the corresponding altered scenario, in which only one working condition is changed. In parenthesis: empirical standard errors

Highest COVID-19 impact	$\overline{\Delta predicted}^{\,j}_{social\,soft\,skills}$
	($$SE_{\overline{\Delta predicted}^{\,j}_{social\,soft\,skills}}$$)
**G19A (Working with computers)**	
G34A *(Managing working groups)*	0.0083 (0.0078)
C16A *(Service orientation)*	0.0081 (0.0086)
G37A *(Making people grow)*	0.0076 (0.0073)
**H1 (Face-to-face discussions)**	
C32A *(Time management)*	$-$0.0054 (0.0068)
G38A *(Consultancy)*	$-$0.0086 (0.0080)
C35A *(Human resources management)*	$-$0.0120 (0.0074)
**H8 (Dealing with external customers)**	
G35A *(Training)*	0.0015 (0.0077)
G38A *(Consultancy)*	0.0006 (0.0081)
F6 *(Taking care of others)*	$-$0.0016 (0.0084)
**H21 (Physical proximity)**	
G36A *(Guiding, directing and motivating the subordinates)*	0.0023 (0.0068)
C14A *(Negotiating)*	$-$0.0001 (0.0075)
G38A *(Consultancy)*	$-$0.0005 (0.0081)
**H29 (Exposure to disease/infections)**	
F6 *(Taking care of others)*	0.0103 (0.0085)
F7 *(Teamworking)*	0.0036 (0.0082)
C16A *(Service orientation)*	0.0030 (0.0087)

$^*$Statistically different from 0 at the 95% confidence level

**Table 8:** Three social soft skills with the lowest empirical mean variation — averaged over the Italian working population and also over the three simulated post-COVID-19 scenarios — of the MC prediction for each single altered condition. Negative values imply a deficit of social soft-skills endowment associated with the corresponding altered scenario, in which only one working condition is changed. In parenthesis: empirical standard errors

Lowest COVID-19 impact	$\overline{\Delta predicted}^{\,j}_{social\,soft\,skills}$
	($$SE_{\overline{\Delta predicted}^{\,j}_{social\,soft\,skills}}$$)
**G19A (Working with computers)**	
C11A *(Social perception)*	$-$0.0147 (0.0080)
F4 *(Leadership)*	$-$0.0128 (0.0069)
C13A *(Persuading)*	$-$0.0081 (0.0089)
**H1 (Face-to-face discussions)**	
C15A *(Teaching)*	$-$0.0274$^*$ (0.0076)
C16A *(Service orientation)*	$-$0.0242$^*$ (0.0087)
G34A *(Managing working groups)*	$-$0.0233$^*$ (0.0078)
**H8 (Dealing with external customers)**	
F4 *(Leadership)*	$-$0.0246$^*$ (0.0069)
G36A *(Guiding, directing and motivating the subordinates)*	$-$0.0217$^*$ (0.0068)
F7 *(Teamworking)*	$-$0.0181$^*$ (0.0082)
**H21 (Physical proximity)**	
F7 *(Teamworking)*	$-$0.0337$^*$ (0.0082)
C12A *(Coordination with others)*	$-$0.0267$^*$ (0.0063)
F5 *(Cooperating)*	$-$0.0264$^*$ (0.0054)
**H29 (Exposure to disease/infections)**	
G35a *(Training)*	$-$0.0066 (0.0077)
G36A *(Guiding, directing and motivating the subordinates)*	$-$0.0063 (0.0069)
C35A *(Human resources management)*	$-$0.0058 (0.0074)

$^*$Statistically different from 0 at the 95% confidence level

It is worth observing that some social soft skills repeatedly occur in either Table 7 or 8, and that some of them also appear in Table 4. More in detail, the obtained results show that when considering a single changing column associated with one of the five selected working conditions one often finds among the most affected social soft skills, the same social skill for different choices of that column: in particular, consultancy (G38A) is associated with one among the three highest empirical mean variations in the predicted level in 3 of the altered scenarios considered in Table 7, while teamworking (F7) experiences one of the three lowest empirical mean variations in two of the altered scenarios considered in Table 8. These two social skills appear also in Table 4. It is worth remarking that, when performing the robustness checks (i.e., when moving from Table 4 to Tables 7 and 8), several of the results associated with the lowest COVID-19 impact continued to be statistically significant. Precisely, this occurred in the case of the following altered conditions: face-to-face discussions (H1), dealing with external customers (H8), and physical proximity (H21). The robustness of such results turns out to be particularly relevant, given that these are working conditions that are reasonably expected to change in the future in view of the increasing diffusion of remote working in the post-COVID-19 phase.

## Final discussion

In this work, we exploited similarities in the Italian occupational structure and implemented a recent machine-learning technique (namely, matrix completion) to predict the average importance levels of social soft skills employed in each occupation and to identify the needs for such social soft skills in occupations by examining deficits and surpluses in social soft-skills endowment associated with changes in the working conditions induced by COVID-19. Our matrix completion analysis was accomplished at the level of each profession (only in a successive step, the results were aggregated at different levels for better visualization and interpretation). More precisely, in our analysis, matrix completion was applied several times, with different selections of the training/validation/test sets. In each such application, a specific row (profession) was chosen in such a way that the test set was extracted from that row. Moreover, each row was associated with the test set in several different applications of matrix completion, which allowed us to get and analyze its performance statistics for each specific row. Concluding, by proceeding in this way, our analysis implicitly took into account the possible dependence on the profession of the average importance level of each social soft skill.

The first part of our analysis, based on matrix completion, was made at the level of each profession/social soft skill (which refer, respectively, to the indices *i* and $j \in J$ in the notation used in Sect. 4.2). Then, results were aggregated at different levels in the successive part of the analysis, as explained in Sect. 4.2. This aggregation was done to provide an interpretable higher-level analysis and also to increase the likelihood of obtaining statistically significant results. Indeed, a limitation of the present work is that the current application of matrix completion is computationally intensive,^29^ which limits the amount of numerical results that can be obtained with a reasonable computational effort considering all possible positions in the test set, each of which corresponds to a specific pair “profession/social soft skill”. In fact, in our study, the total number of such pairs considered in the analysis was $796*21=16\,716$. A more detailed analysis of a subset of suitably-selected pairs “profession/social soft skill” is left for possible future research.

In our analysis, we considered three possible scenarios for the impact of COVID-19 (together with some robustness checks), yet our results could give us a preliminary insight into trends in the labor market in the near future. Limiting the discussion to results that turned out to be statistically significant, among social soft skills, we report the largest deficits induced by the simulated post-COVID-19 scenarios for cooperating (F5), managing working groups (G34A), coordination with others (C12A), teamworking (F7), and teaching (C15A), whereas we find a surplus only for consultancy (G38A). Results related to the largest deficits turned out typically to be statistically significant also when performing robustness checks. Precisely, this occurred in the case of altered conditions related to face-to-face discussions (H1), dealing with external customers (H8), and physical proximity (H21). The robustness of such results turns out to be a particularly relevant outcome since they refer to working conditions that are reasonably expected to change in the future as a consequence of the increasing adoption of remote working in the post-COVID-19 scenario. Moreover, our results suggest that wholesale and retail trade, accommodation and catering services, education, healthcare and social services, and other service activities, suffered the largest deficits in social soft-skills endowment due to changes in working conditions. On average, the age groups under 35 years old were more negatively affected by the simulated changes in working conditions than the older age groups.

When the COVID-19 pandemic is over, it will be of primary interest for the public and the business sectors to formulate effective alternatives to maintain and promote the favorable mutations in labor markets that the crisis has provoked. A composite and hybrid working model might become dominant, e.g., a model in which workers can decide whether to work at the office or from home, even blending these two conditions during the working week. In accordance with the content of tasks, together with personal needs or preferences, employees and managers will be asked to find new working conditions that merge the advantages of direct personal and physical contact with the flexibility of teleworking. Definitely, smart-working is not suitable for everyone and the quantity of smart-working adopted during the pandemic might have been disproportionate. A balance between employers’ and employees’ preferences is desirable, as well as some minor changes in the organization of work. This consideration has to be tackled by policymakers after the lesson we learned from the COVID-19 pandemic: there is an unexploited capability, which might result in a gain in efficiency for employers and employees who are willing to work from home more if frictions in the national legislation and internal organization of workplaces are addressed. All those changes related to digitalization, artificial intelligence, smart working, and the platform economy go hand in hand with reinforcements in occupational health and safety, social security systems, and workers’ rights (European Council Porto Declaration, May 2021). The pandemic has been a stress test, in the sense that it has highlighted where to invest more in order to improve connectivity or upskilling of workers, and at the same time it has dismantled psychological and cultural barriers to smart working, as it has obliged both employers and employees to win their previous reluctance about smart-working, and in fact, they are now expressing preferences for higher shares of teleworking hours with respect to pre-pandemic levels. Our results suggest which are the social soft skills that required an update and upgrade in the labor market. Social soft skills, in fact, might need some time to adjust to these rapid changes in the organizational structure and in the labor market, thus workers may need to undertake specific and tailored training that goes in this direction. Finally, in our specific application, matrix completion has demonstrated an excellent prediction capability, as well as making us able of carrying out a counterfactual analysis of pre- and post-COVID-19 occupational structure. The path for future research is wide as more data will be made publicly available at the end of the pandemic, so as to confront these results with the actual values. The methodology proposed in this article could also be applied to examine other recent trends in the labor market, and possibly also to make a forecast analysis for future trends. To conclude, our research does not exhaust the possible analyses that can be performed based on the ICP dataset, possibly focusing on other variables of interest. For instance, in Gnecco, Landi, and Riccaboni (2022), we analyzed the average importance levels of soft skills related to creativity. Other possible future analyses could be focused, e.g. on the relationship between social soft skills and digital skills of workers.^30^

## Data Availability

The data used for this work are available for research purposes at the following hyperlinks: (a) Indagine Campionaria sulle Professioni (ICP): https://inapp.org/it/dati/ICP; (b) Microdata For Research (MFR) on the Continuous Detection of Labor Force (RCFL): https://www.istat.it/it/dati-analisi-e-prodotti/microdati; c) ISTAT data on the Italian population: http://dati.istat.it/index.aspx?QueryId=18460 &lang=en#.

## Footnotes

1 Details about the design of the survey (particularly about its specific questions) are available at https://inapp.org/it/dati/ICP.

2 More precisely, we formulate and solve an MC optimization problem (see Eq. (1) in Sect. 4.1) whose optimal solution allows one to predict optimally elements in specific columns (related to social soft skills) of each modified ICP matrix, based on a subset of other elements of that matrix and a suitable regularization of the resulting reconstruction (or completion) of the modified ICP matrix. The regularization term aims to prevent overfitting.

3 The acronym is because of the original Italian denomination: “Rilevazione Continua sulle Forze di Lavoro.”

4 The COVID-19 pandemic originated from the town of Wuhan in China at the end of 2019 and spread quickly across the world, reaching about 213 countries (Roser et al. 2020). Its spillover effects have been devastating, particularly the ones related to deaths and job losses. To contrast the spread of the virus and contain the burden on the respective healthcare systems, most governments around the world enforced lockdowns and quarantines, i.e., restrictive physical and social distancing countermeasures that also raised concerns related to their negative effects on the economy and on social well-being. For instance, the COVID-19 pandemic has had a quite severe impact on employees, employers, graduates, and the labor market in general.

5 Furthermore, at the time of the lockdowns, governments required the majority of non-essential businesses to close, impacting negatively national economies and leading to a significant drop in employment (Khalid et al. 2021, Pieroni, Facchini, and Riccaboni 2021). More generally, the COVID-19 pandemic has had an impact on the quality of work life (Majumder and Biswas 2022). While many of the implications of the lockdown on the economy were negative, there was also some positive progress as firms were forced to adapt to a “new normality”. Now banks are dealing with higher credit risks than before, while insurance companies are expanding their digital assets. Some traditional office-based businesses experienced significant cost reductions by shifting to remote working, while restaurants and bars moved towards takeaway and delivery services.

6 The European Union Labor Force Survey (EU LFS), started by Eurostat in 1983, is a large household survey that provides statistics on labor participation. It is based on several national surveys, which are conducted by the national statistical institutes of the member states. Such institutes are also responsible for the sample selection, the preparation of the questionnaires and the interviews. Results are then forwarded to and assembled by the European institute. The EU-LFS covers all industries and occupations.

7 These data are provided for research purposes by INAPP at the following hyperlink: https://inapp.org/it/dati/ICP.

8 The ATECO (ATtività ECOnomiche) classification of economic activities was adopted in 2007 by the Italian National Institute of Statistics (ISTAT) for the national statistical summaries on the economic landscape. It is the translation of the Eurostat NACE Rev. 2.

9 See, respectively, the following hyperlinks: https://www.istat.it/it/dati-analisi-e-prodotti/microdati, http://dati.istat.it/index.aspx?QueryId=18460 &lang=en#.

10 The International Standard Classification of Occupations (ISCO) is an International Labor Organization (ILO) classification structure for organizing information related to labor and jobs. ISCO is defined by ILO itself as a tool aimed to organize jobs into a properly constructed set of classes according to the tasks and duties specific to each job. The last classification was adopted at the end of 2007 and is known as ISCO-08.

11 Details on the sample stratification can be found in the file “Nota metodologica”, available at the following hyperlink: https://inapp.org/sites/default/files/ICP_nota%20metodologica_0.pdf. The document also describes how the issue of possible non-responsiveness is solved by adopting several reserve lists for each profession.

12 It is worth noting that the ICP dataset is characterized by the following features: (1) accuracy, granularity, and richness; (2) specificity to the Italian productive system. In this way, one possibly avoids biases occurring when information related, e.g. to the US occupational structure is linked to labor market data related to different economies such as the European ones. More information on this issue is provided in Bonacini et al. (2021) and Vannutelli et al. (2022).

13 The reason behind this reclassification is evident from Table 1: the three items “Coordination with others”, “Cooperating”, and “Coordinating”, are highly related to each other and refer to social soft skills, although they were originally classified (according to the ICP survey design) in three different macro-categories (“Competencies”, “Working styles”, and “Generalized working activities”).

14 The ICP dataset provides actually also some additional information, as the survey also explores, e.g. the average intensity level in the use of each skill, conditional on a sufficiently high importance level expressed by the participant for that skill. In order to simplify our application of matrix completion, only the average importance levels have been taken into account, ending up in a matrix of smaller size, whose entries are also simpler to interpret.

15 This expectation is justified by the fact that already the original ICP matrix is well-approximated by a low-rank matrix (i.e. its singular values decay rapidly to 0), as highlighted in Gnecco, Landi, and Riccaboni (2022). This holds also for its perturbations considered in the present study. As discussed in Gnecco, Landi, and Riccaboni (2022), a rapid decay to 0 of the singular values of a matrix is an important prerequisite for a successful application of matrix completion to such a matrix.

16 The analogous figure with the 21 ATECO sections replaced by the 88 ATECO sectors is not reported, due to space reasons.

17 Since the MFR RCFL dataset exploits profession codes at the 4-digit level, whereas ICP considers profession codes at the 5-digit level, in order to compute the percentages reported in Fig. 3a and b, it has been assumed that profession codes at the 5-digit level which correspond to the same profession code at the 4-digit level are characterized by the same number of workers. Moreover, in order to get the percentage of Italian workers associated with each profession, information about the Italian population in each Italian region (in 2020) has been taken into account (source: http://dati.istat.it/index.aspx?QueryId=18460 &lang=en#). More precisely, first, the percentage of Italian workers in 2020 associated with each profession has been determined for each region, based on the MFR RCFL dataset, averaging over the 4 quarters of that year. Second, the percentage of Italian workers in 2020 associated with each profession has been determined for the whole of Italy in the same year, taking into account the amount of the Italian population in each Italian region in the same year. This has been performed both for each age group and for all the age groups simultaneously.

18 Further details on the specific application of MC are similar to those reported more extensively in Gnecco, Landi, and Riccaboni (2022).

19 This can be interpreted as the product of a loading matrix and a factor matrix.

20 This scenario and this percentage have been chosen to generate the figure because they represent intermediate cases, respectively among the three scenarios and among the three percentages considered in the analysis.

21 For each profession, the (empirical) mean and standard deviation were computed with respect to the repetitions having as test set elements belonging only to the row of the matrix which is associated with that specific profession.

22 One can notice from Fig. 5 that, for the specific case reported therein, the MC performance on the test set turned out to be slightly better than its performance on the validation set. This could be explained by observing that the elements of these two sets came from different portions of the occupation matrix. Besides, the test set had a much smaller number of elements than the validation set because the former elements were associated with a specific row of the occupation matrix.

23 When considering its realizations (indexed by *r*), a second average was taken with respect to *r*, to evaluate the empirical mean, likewise in Eq. (3).

24 This was justified by the large number of (approximately) independent random variables in each weighted average.

25 For the cases in which the estimated empirical standard errors were much smaller than the corresponding empirical means, the results are likely to be robust with respect to the fact that the standard deviations were estimated assuming independence of the random variables constituting the weighted averages.

26 It is worth noting that also the results obtained for the other social soft skills not reported in Table 4 turned out to be statistically significant.

27 It is worth noting that also most of the results obtained for the other ATECO sectors not reported in Table 6 turned out to be statistically significant.

28 This is not the case only for the ATECO section N (Rental, travel agencies, business support services), for which, however, the empirical mean variation of the MC prediction was very low in absolute value, compared to the other ATECO sections.

29 One future extension of the work could consist in speeding up its matrix completion analysis by using, e.g., parallel computing.

30 It is worth noting that one of the columns of the ICP matrix refers to the average importance level of computer skills for each profession (ICP item code: B9A). So, in our analysis, matrix completion also took into account that column for prediction purposes, although identifying its specific contribution to the predictions would require additional analyses, which are left for possible future research.
